# Impact of Drill Bit Wear on Screw Withdrawal Resistance in Pinewood

**DOI:** 10.3390/ma17235729

**Published:** 2024-11-23

**Authors:** Maciej Sydor, Krystian Waszkiewicz, Zbigniew Potok

**Affiliations:** 1 Department of Woodworking and Fundamentals of Machine Design, Faculty of Forestry and Wood Technology, Poznań University of Life Sciences, 60-637 Poznań, Poland; 2 Department of Furniture Design, Faculty of Forestry and Wood Technology, Poznań University of Life Sciences, 60-637 Poznań, Poland; zbigniew.potok@up.poznan.pl

**Keywords:** furniture, woodworking, brad point bit, pilot hole, drilling, screw pull-out resistance, axial withdrawal of screws, wood screws, wood

## Abstract

Many factors affect screw withdrawal resistance (SWR), including screw size, embedment depth, the pre-drilled hole’s diameter, dimensional accuracy, and the furniture pieces’ material properties being joined. While prior research has extensively examined the influence of these factors, this study aimed to explore a neglected factor: how drill bit wear impacts pilot hole quality and subsequent SWR. The experimental setup included pinewood samples with pre-drilled 5 mm diameter blind pilot holes with a depth of 45 mm. The holes were equally divided into two groups: one drilled with a sharp bit, the other with a blunt bit. Euro-type coarse furniture screws (7 mm major diameter, 4 mm minor diameter, 3 mm pitch) were screwed into all holes. Subsequently, SWR was measured using a universal testing machine. Results show a statistically significant decrease in SWR when using the blunt drill bit. This phenomenon can be explained by excessive local material degradation, increased surface roughness, and disrupted hole dimensional accuracy, collectively hindering SWR. The study’s findings offer insights into how excessive drill bit wear impacts the screw withdrawal capacity of pinewood, informing best practices in furniture and construction.

## 1. Introduction

Wood screws are widely used as fasteners in furniture and construction applications. Screw withdrawal resistance (SWR) is a measure of a screw’s ability to resist the pulling force required for its removal from wood. The SWR value primarily influences the efficient load-bearing capacity of joints employed in wooden products. A high SWR enhances the overall reliability of furniture and building structures, so this parameter is crucial in engineering design [1]. SWR is often measured experimentally to compare the performance of various wood-based engineered materials [2]. Wood screws can also be used as probes to quantify a semi-destructive evaluation of a wood’s structural degradation during service time of wooden structures [3].

The primary approach to ascertain the SWR value is through an empirical test, followed by extrapolation using established equations that incorporate screw design and material properties [4,5,6]. The scholarly literature suggests that the SWR of pinewood is impacted by the following: screw size, screwed depth and thread design [4], the wood material’s inherent characteristics [7], the orientation of screw insertion related to the wood grain [8], the technique of screw insertion, including screwing torque used [9], and the distance of the screw from the edge of the wooden element [10]. Previous research has also demonstrated that cyclic temperature fluctuations, such as those experienced during seasonal changes, can significantly impact the mechanical properties of wood, including its resistance to screw withdrawal. Specifically, it has been observed that freezing (−25 °C) and heating (+70 °C) cycles lead to slight decreases in SWR, while heating cycles result in increased SWR [11]. However, significantly increasing the modification temperature, above 190 °C, reduces the SWR [12]. These effects are attributed to wood microstructure changes [13]. The other studies indicate that SWR is not correlated with the width of annual rings in the tree trunk, suggesting that this specific wood attribute does not influence SWR [14]. In contrast, the parameters involved in drilling pilot holes impact the SWR value. A high drilling speed during pilot hole creation notably adversely affects SWR [15,16].

Despite extensive research on SWR, the impact of drill bit bluntness on pinewood remains underexplored. This research addresses this gap, as worn drill bits are commonly used in industrial settings. There is a common practice of using drill bits beyond their optimal lifespan in industrial settings [17]. This practice, driven by cost considerations, can delay replacing worn-out bits, often due to a lack of adherence to strict maintenance schedules or operator oversight. This is despite exceeding the drill’s lifespan, ultimately increasing machining costs [18].

This study investigates the hypothesis that the sharpness of the drill bit used to create pilot holes influences the force necessary to extract screws from pinewood.

## 2. Materials and Methods

### 2.1. Materials

#### 2.1.1. Pinewood Description

The experimental setup included pinewood, a budget-friendly softwood commonly used in European furniture production, especially for bed frames, sofas, and chairs. Pinewood’s ease of cutting, shaping, and drilling contributes to efficient construction. While softer than some hardwoods, pinewood offers adequate strength for furniture frames [19,20]. Pine’s lightweight nature also simplifies handling during manufacturing, assembly, or moving the finished furniture.

Sixty sapwood cubes were prepared from European pine (*Pinus sylvestris* L.) as test samples. Each block measured 50 mm on each side. The cubes were then randomly divided into two series of 30 each, labeled Series A and Series B. To ensure consistent moisture content (MC) across all the pinewood blocks, they were stored indoors under identical environmental conditions for three months (RH = 65 ± 5%, t = 20 ± 3 °C). The moisture content and density of the samples were measured immediately after the screw withdrawal tests using an oven-dry method and calculated as follows:

$$MC=\frac{m_{m}-m_{o}}{m_{o}}\cdot 100$$

where *m*_m_ was the block mass, and *m*_o_ was the oven-dry mass of the block. Moisture content (MC) was determined by measuring the mass and oven-dry mass of two specimens, one from each series. Each weighing was performed three times, and the average of the three measurements was recorded. The density was calculated based on the dimensions and mass of the ten samples in each series. Table 1 presents the results for MC and average density calculations.

The weight of the samples was measured with an electronic laboratory balance (model PA 213/1, OHAUS, Parsippany, NJ, USA), with a measurement uncertainty of Δ_m_ = ±0.001 g. Sample dimensions were measured using calipers accurate to ±0.05 mm. The volume for average density calculations was calculated by subtracting the nominal hole volume from the dimensions.

The study utilized clear wood samples only to ensure that defects did not influence our results in the wood material.

#### 2.1.2. Screws

**Figure 1:**
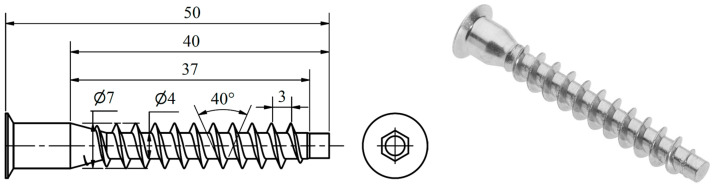
Screw used in the SWR measurements.

The experimental setup included 60 Euro-type screws (the so-called confirmat screws), popular in furniture assembly, especially in ready-to-assemble furniture. Their coarse, deep threads compress the material, forming an internal thread as a grip within the 5 mm pre-drilled pilot hole. These screws, with the 5 mm pilot hole and 8 mm clearance hole made in furniture elements, align with “system 32”, a widely used standard in furniture made of wood-based panels [21]. Figure 1 shows the screw used.

#### 2.1.3. Drill Bit

The experimental setup included two industrial 5 mm twist brad drills: a new, fully sharp, and a blunt one. Both were of the same type (with a total length of 70 mm, nominal cutting circle diameter of 5 mm, cutting length of 35 mm, helical pitch of 17.5 mm, cylindrical shank dimension 10 × 20 mm with milling, right-hand rotation, blade made of HM carbide, two cutting edges, two peripheral cutters (WN1.050.035.070.00R, ITA Tools, Kraków, Poland). These drills were intended to make blind pilot holes in the pinewood samples. The blunt drill employed in this study had been extensively used in producing wooden and wood-based furniture components, drilling approximately at least 1200 holes before this research (according to Czarniak et al. study, it can be concluded that such drills typically wear by about 90% after creating 600–700 holes and become entirely blunted after approximately 1100 holes [17]).

Drill wear is a random process, making it difficult to predict when and how it will occur [22]. The subjective nature of assessing drill bit bluntness results in inconsistent practices in industrial settings, leading to premature and delayed replacement of drill bits. Consequently, the assumption that a drill is blunt after drilling 1200 holes is a conventional estimate adopted for the needs of this study.

**Figure 2:**
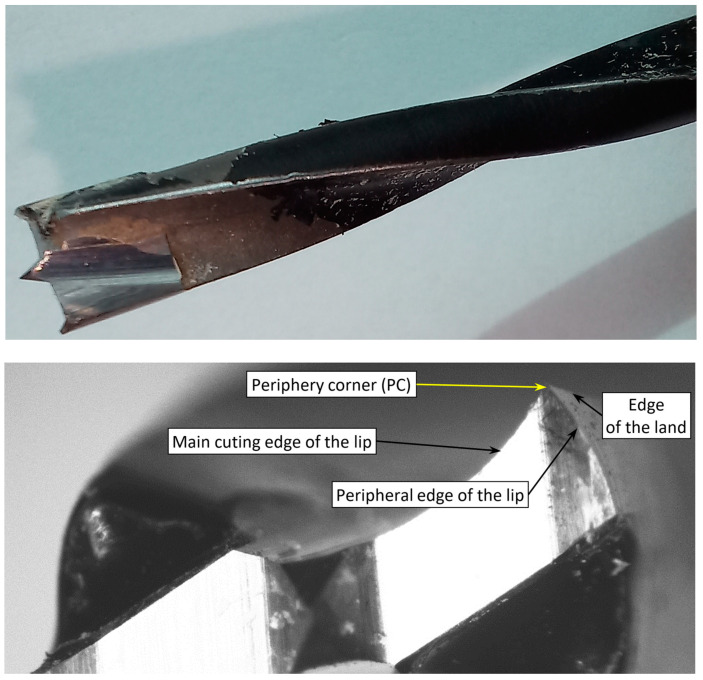
The twist brad drill used.

Figure 2 shows that the cutting elements of the twist drill bit used in the study contain two periphery corners formed by three edges: the primary cutting edge of the lip, the peripheral edge of the lip, and the screw-shaped edge of the land.

A brad point drill bit is a specialized tool designed for drilling in wood and wood-based materials in industrial conditions. Its W-shaped tip has a center point, two cutting edges, and two peripheral cutters. The center point prevents the drill bit from wandering when the hole-making starts. Two cutting edges with two peripheral cutters create the main cutting action, producing an internal surface of the hole. The peripheral cutters prevent the wood material from splintering when drilling through the workpiece. Additionally, the brad point drill bit cuts a circular groove around the periphery of the hole before the cutting edges plane the bottom. This allows for a cut of the fibers, preventing them from being pulled out and compromising the hole’s quality.

## 3. Methods

### 3.1. Drilling and Screwing the Screws

**Figure 3:**
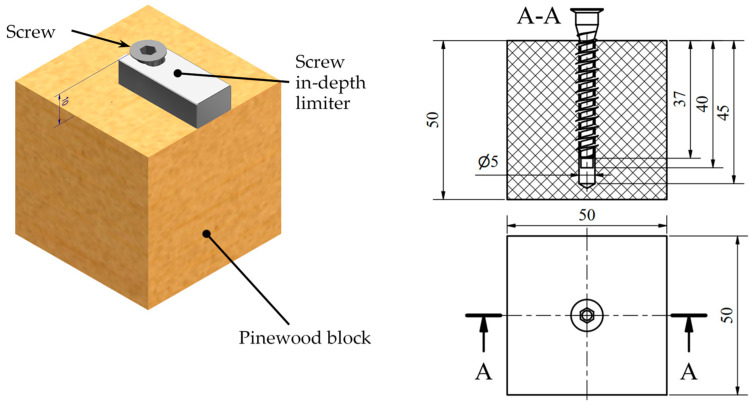
Test samples for screw withdrawal resistance measurements.

Each cube-shaped pinewood block used in the study received a blind hole drilled using an industrial CNC machine (Creator 950, Felder Group, Hall in Tirol, Austria). These holes were 5 mm in nominal diameter and 45 mm in nominal depth and were drilled along the wood grain (longitudinal direction of the stem). The machine operated with cutting parameters following the tool manufacturer’s recommendations [23] at a spindle speed of 4500 rpm and a feed rate of 2.0 m/min, resulting in a cutting speed of 0.45 mm/rev and a drilling time of 1.3 s. Holes in the Serie A of test samples were drilled with a sharp drill bit, while holes in the Serie B were drilled with an entirely blunt drill bit. The Euro-type screws were screwed to a depth of 40 mm using the jig as a screw-in-depth limiter (Figure 3).

**Figure 4:**
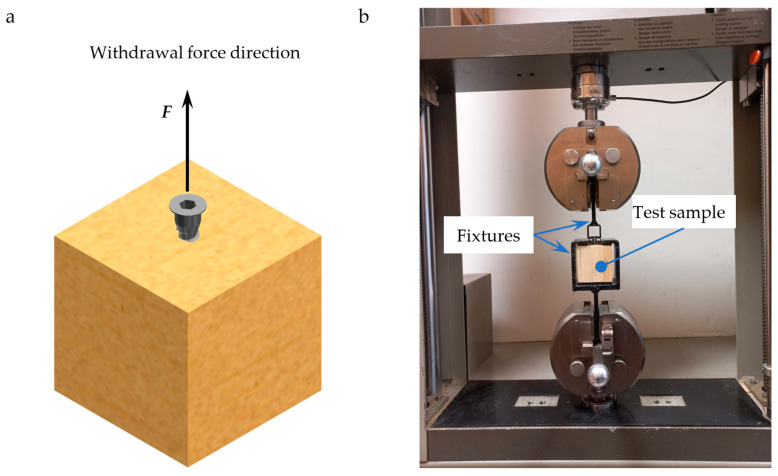
Experimental setup: (**a**)—test sample; (**b**)—withdrawal measurements.

As shown in Figure 4, the screw-withdrawal resistance (SWR) was measured using a laboratory universal testing machine (model Z050, Zwick Roell Group, Ulm, Germany). The test method was based on the EN 320 [24] standard, with modifications to accommodate the use of a screw commonly used in furniture production. The test samples were fixed in a holder, and the screws were withdrawn at a constant rate of 5 mm/min. During the experiment, an initial tension force of 5 N was applied to the measuring system.

New and blunt drill bits underwent microscopic photography and measurement to identify the nature of the drill bit dulling. Microscopic images of the sharp and blunt drill bits were obtained using a stereomicroscope (Motic SMZ 168 Series, Motic, part of McAudi Corporation, Xiamen, China) equipped with a digital camera (Moticam 5+ camera, Motic). The microscope was interfaced with a computer image analysis system for capturing and analyzing images.

### 3.2. Statistical Analysis

We employed a *t*-test at a significance level of α = 0.01 to assess the statistical significance of the observed differences in SWR. Before the analysis, Chauvenet’s criterion was applied to identify and exclude potential outliers from the experimental dataset. Additionally, we compared both the mean and median values of the two series to understand the central tendency and variability of the data. All calculations were performed in a spreadsheet editor (MS Excel, v. 2312, Microsoft, Redmond, AW, USA).

## 4. Results and Discussion

**Figure 5:**
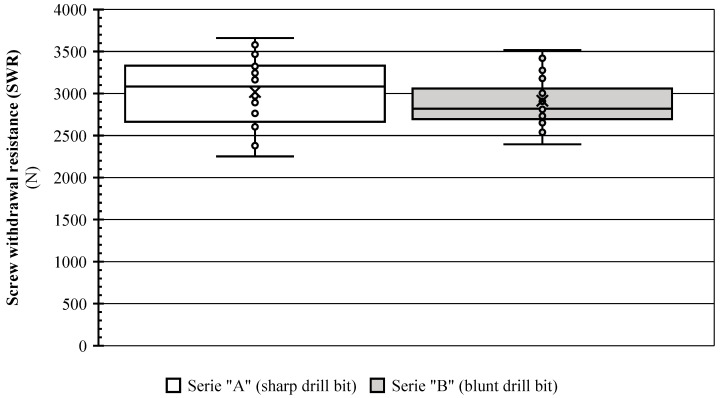
A box plot comparing screw withdrawal resistances in the series (*n* = 28 for each series).

Figure 5 presents a boxplot summarizing the distribution of withdrawal forces measured during the experiment. The mean in the series is marked with an “x” and the median with a horizontal line. The median and mean indicate central tendency, while the box’s interquartile range (IQR) represents data dispersion. Whisker length reflects distribution skewness.

Samples with pilot holes drilled using a sharp drill exhibited significantly higher SWR values, averaging 3018.1 N, compared to those drilled with a blunt drill, which averaged 2914.7 N. The difference in medians is even more significant (3084 N vs. 2820 N). Table 2 summarizes the descriptive statistics of the SWR measurement results.

Outliers were identified and removed from both series of measurement data using Chauvenet’s criterion (four outliers were removed, two from each series). Since the data were free of outliers, a *t*-test was used to compare the means of the two series with a significance level of 0.01. The *t*-statistic is −0.311, which indicates a slight difference in means, with Series A having a lower average value. The *p*-value is 0.755, more significant than the chosen significance level of 0.01. Since the *p*-value (0.755) is greater than the significance level, we fail to reject the null hypothesis that there is no significant difference between the means of the two series. In other words, at a 0.01 significance level, there is insufficient evidence to conclude that Series A and Series B have statistically different average values.

The statistical analysis points with 99% confidence that the mean screw withdrawal force values are significantly different between series. In other words, screws inserted into pilot holes drilled with a blunt drill bit exhibit a lower average withdrawal force compared to those screwed into holes drilled with a sharp drill bit.

**Figure 6:**
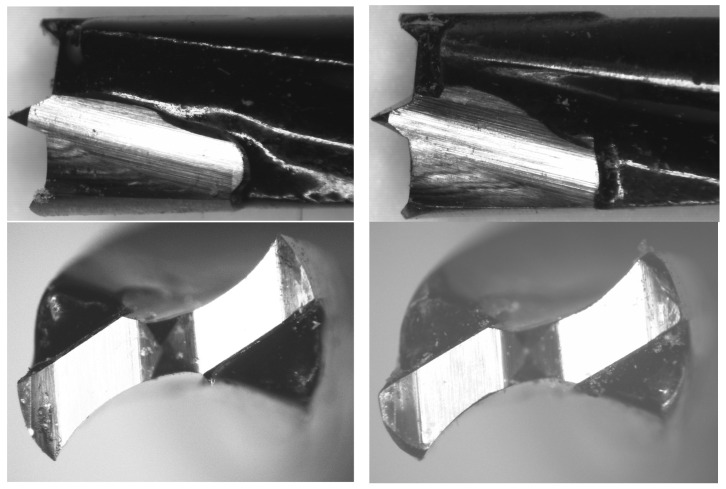
Comparison of drill bits: sharp (**left**) and blunt (**right**) at 10× magnification.

**Figure 7:**
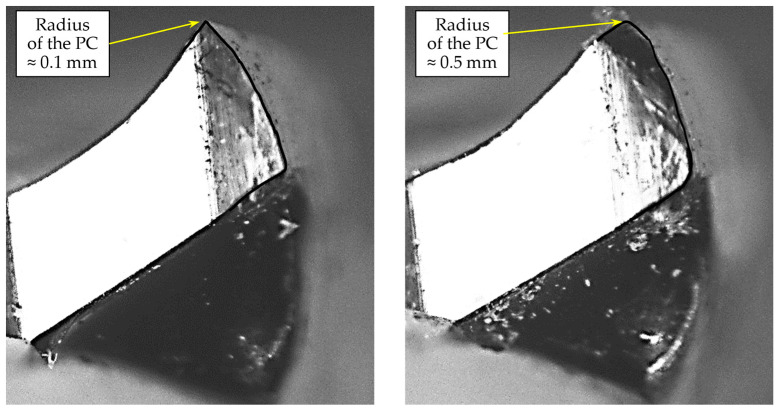
The rounded peripheral corner in sharp (**left**) and blunt (**right**) drill bit (18× magnification).

The experiment results demonstrate that the condition of the drill bit is a critical factor affecting screw withdrawal resistance in screws inserted into pre-drilled pilot holes. Figure 6 and Figure 7 compare the state of all cutting edges and the tip radius in sharp and blunt drill bits used. The drill on the left in the figures is sharp, while the one on the right is blunt.

The most striking difference between the sharp and blunt drill bits is the pronounced rounding of both periphery corners (PC) on the blunt bit. This rounding of PC has increased roughly fivefold, from around 0.1 mm to around 0.5 mm (Figure 7). The blunt drill bit also exhibits minor nicks along its cutting edges.

The terms “sharp bit” and “blunt drill bit” are blurred in woodworking, but the difference is palpable. Both terms refer to the condition and effectiveness of drill bits used to make holes in wood and other materials. A sharp drill bit has clean, well-defined cutting edges and a pointed tip. It easily cuts into the wood, creating smooth, precise holes with minimal energy expenditure. Sharp bits remove wood without excessive pressure and produce less tear-out, which is when the wood fibers around the edge of the hole splinter or fray. Blunt bits struggle to cut effectively, require more force, and generally create rougher holes. They tend to generate more heat due to friction, damaging both the bit and the wood material. Studies have shown that blunt drill bits lead to increased resistance, requiring more feeding force and generating higher friction, which can deteriorate precision in wood density evaluation [25]. Monitoring drill bit sharpness improves the precision of drilled holes and the overall longevity of the drill bit [26].

Drilling pilot holes can induce residual strain, leading to a deterioration of the mechanical properties of the wood material near the hole. This statement is consistent with the research results of other authors. Tang et al. [27] investigated the influence of feed rate during drilling on the residual stress levels in a chosen softwood species. The cited authors observed that higher drilling speeds increased the maximum residual compressive strain. It appears that both the increased feed rate and the deterioration of drill properties (blunting) necessitate a greater force to be exerted during the drilling process. This creates compressive strain, delaminating the internal surface of the hole.

**Figure 8:**
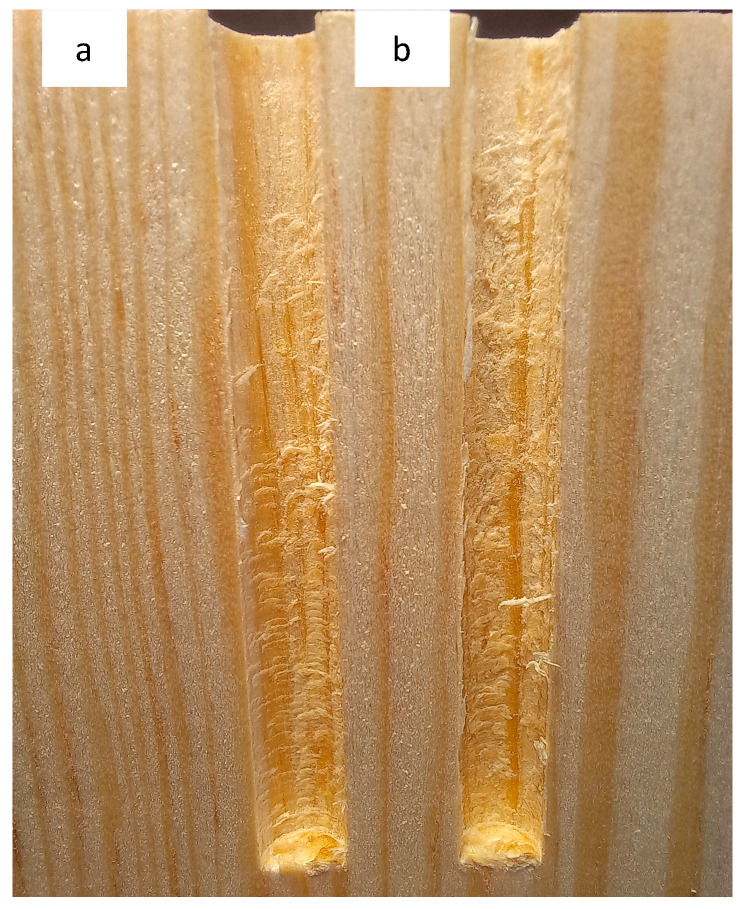
The comparison of holes: (**a**)—made with the sharp drill; (**b**)—made with the blunt drill.

Wood material-specific properties influence susceptibility to delamination, but it is inevitable [28]. Figure 8 illustrates that localized tensile stress exceeding the material’s limits results in surface delamination, characterized by a noticeably rougher surface. Such a surface worsens SWR.

Our study confirms that the drill bit’s condition affects the hole’s overall quality. A sharp drill bit creates a sharper and cleaner hole edge, while a blunt drill bit results in a slightly blurred or ragged edge. Two samples were sectioned and photographed to compare hole surfaces (Figure 8).

Figure 8 illustrates that while all drilled holes exhibit visible surface roughness, those created by blunt drills are notably more pronounced. This increased surface irregularity likely contributed to the lower force required for screw extraction during the experiment. These findings align with previous studies. Drilling with the blunt drill bit likely influences the size and shape of chips produced during drilling, worsening the drilling conditions. A smaller cutting-edge radius in sharp tools tends to generate smaller in size chips, while a larger radius produces larger chips that are more difficult to evacuate through the flutes [29]. As a result, the wear of a drilling tool significantly worsens the quality of the newly formed surface when drilling wood composites [30]. It is important to note that holes drilled in wood, even with a sharp drill and optimal processing conditions, are never perfectly cylindrical, and their shape hangs over time. Sydor et al. [31] studied the influence of changes in humidity on the shape of holes drilled in pine wood samples. The study found that the shape of the drilled hole is an irregular cone with a larger diameter at the bottom. The authors explained this phenomenon by the influence of chips that distort the hole during evacuation through cylindrical drill bit groves.

The presented study results have the following three potential limitations:

1. The study focused on pinewood, and these findings may not be directly transferable to all wood species.
2. This study employed a standard industrial drill and manufacturer-recommended parameters. However, variations in machine tooling, drill type, and machining conditions (rotational speed, feed rate) could influence the observed results.
3. We used a 5 mm pilot hole and a standard furniture euro-type screw. Other screw-pilot hole dimensional combinations may result in different results (especially since the screw withdrawal force is very strongly correlated with the pilot hole diameter).

Regardless of these limitations, the study results offer evident practical implications highlighting the importance of controlling the drill wear during pilot hole drilling to achieve optimal screw withdrawal resistance.

## 5. Conclusions

Test results demonstrate that drill bit wear diminishes screw withdrawal resistance (SWR) in pinewood samples. The observed differences in SWR are attributed to the quality and precision of the pilot holes drilled with sharp versus dull bits. Three factors can explain this:

- The use of sharp drill bits results in precise pilot holes, facilitating the secure engagement of screw threads with wood fibers and enhancing withdrawal resistance. In contrast, dull bits can produce potentially oversized holes due to increased force and vibration, leading to reduced grip between the screw and wood and, consequently, lower SWR.
- Worn drill bits generate larger chips, which can impede chip evacuation and lead to hole deformation. This results in damage to the interior surface of the hole, lowering SWR.
- Dull drill bits generate excessive heat, charring the wood around the pilot hole and reducing friction between the screw and wood, leading to weaker SWR.

Sharp drill bits maintain precise hole dimensions and preserve the integrity of the surrounding wood fibers, resulting in superior SWR compared to dull bits. These findings contribute to more efficient and sustainable construction practices and enhance the quality and longevity of wood-based products.

## Figures and Tables

**Table 1:** Average moisture content (MC) and density of samples.

Sample Serie	Average Moisture Content (%) (*n* = 3, SD in Parentheses)	Average Density (kg/m^3^) (*n* = 10, SD in Parentheses)
A, sharp drill bit	8.60 (0.10)	0.573 (0.016)
B, blunt drill bit	8.70 (0.10)	0.582 (0.018)

**Table 2:** Descriptive statistics.

Parameter	Sharp Drill Bit	Blunt Drill Bit
Mean	3018.1 N	2914.7 N
Median	3084 N	2820 N
Standard deviation	392.4	286.3
Minimum	2252 N	2396 N
Maximum	3660 N	3516 N
Range	1408 N	1120 N
Total number of samples	30	30
Outliers (not included in the statistics)	2	2

## Data Availability

The original contributions presented in the study are included in the article; further inquiries can be directed to the corresponding author.
